# Health Care Workers’ Expectations of the Mercury Advance SMARTcare Solution to Prevent Pressure Injuries: Individual and Focus Group Interview Study

**DOI:** 10.2196/47992

**Published:** 2024-04-18

**Authors:** Joeri Slob, Thijs van Houwelingen, Helianthe S M Kort

**Affiliations:** 1 Nursing Sciences, program in Clinical Health Sciences University Medical Center Utrecht Utrecht University Utrecht Netherlands; 2 Research Group Technology for Healthcare Innovations Research Centre for Healthy and Sustainable Living University of Applied Sciences Utrecht Utrecht Netherlands; 3 Building Healthy Environments for Future Users Group, Department of the Built Environment Eindhoven University of Technology Eindhoven Netherlands

**Keywords:** digital technology, pressure injury, health care professionals, mobile phone, health care workers

## Abstract

**Background:**

The transformation in global demography and the shortage of health care workers require innovation and efficiency in the field of health care. Digital technology can help improve the efficiency of health care. The Mercury Advance SMARTcare solution is an example of digital technology. The system is connected to a hybrid mattress and is able to detect patient movement, based on which the air pump either starts automatically or sends a notification to the app. Barriers to the adoption of the system are unknown, and it is unclear if the solution will be able to support health care workers in their work.

**Objective:**

This study aims to gain insight into health care workers’ expectations of factors that could either hamper or support the adoption of the Mercury Advance SMARTcare unit connected to a Mercury Advance mattress to help prevent patients from developing pressure injuries in hospitals and long-term care facilities.

**Methods:**

We conducted a generic qualitative study from February to December 2022. Interviews were conducted, and a focus group was established using an interview guide of health care workers from both the United Kingdom and the Netherlands. Thematic analysis was performed by 2 independent researchers.

**Results:**

A total of 14 participants took part in the study: 6 (43%) participants joined the focus group, and 8 (57%) participants took part in the individual interviews. We identified 13 factors based on four themes: (1) factors specifically related to SMARTresponse, (2) vision on innovation, (3) match with health care activities, and (4) materials and resources involved. *Signaling function, SMARTresponse as prevention, patient category, representatives,* and *implementation strategy* were identified as facilitators. *Perception of patient repositioning, accessibility to pressure injury aids,* and *connectivity* were identified as barriers.

**Conclusions:**

Several conditions must be met to enhance the adoption of the Mercury Advance SMARTcare solution, including the engagement of representatives during training and a reliable wireless network. The identified factors can be used to facilitate the implementation process.

## Introduction

### Background

In recent years, the world’s population has increased rapidly. In 1950, there were 2.5 billion people on our planet. In 2019, this number had increased to 7.7 billion [1]. Since the 1900s, the global average life expectancy has more than doubled and currently stands at >70 years [2]. Together with the global shortage of health care workers, in particular, nurses and midwives, this results in challenges for patient care [3]. One of the 5 most common injuries experienced by patients is a pressure injury. In 2016, the overall number of patients developing pressure injuries was estimated to be 2.5 million worldwide [4]. Patients with pressure injuries have higher 30-day readmission rates [4], and damage due to pressure injuries can result in complications such as amputation, septic infection, impaired health-related quality of life, and premature death [5]. Along with the global increase in life expectancy, innovation and efficiency in the current health care practices are necessary to preserve the quality of care for patients who are at risk of developing pressure injuries.

Current ways to prevent or treat pressure injuries usually include pressure-relieving devices, wound care, and patient repositioning [6]. For pressure-relieving devices, there are beds, mattresses, and mattress toppers. A subdivide is made between reactive static surfaces (constructed using foam, fiber, air cells, or water bags) that apply constant pressure to the skin and active (alternating pressure) surfaces that regularly redistribute the pressure underneath the body. However, according to a recent Cochrane systematic review [6], there is low-certainty evidence that the alternating pressure of active air surfaces reduces the risk of developing new pressure injuries compared to foam surfaces alone. A combination of reactive static surfaces and active surfaces is called a hybrid mattress, which usually consists of foam and air cells and can be connected to a control unit that is used to power the alternating air function.

The hybrid mattresses provided by Direct Healthcare Group (DHG) were clinically effective in a multisite evaluation study [7] conducted in 8 hospitals in the United Kingdom. DHG recently developed a control unit called Mercury Advance SMARTcare [8]. This unit is used to power the alternating air function on their alternating pressure (active) air surfaces (Mercury Advance mattress). The control unit can be connected to an app that can automatically turn on the alternating air function after a detected period of patient nonmovement. The app can also notify the health care professional of the detected patient’s nonmovement; therefore, the health care professional can turn on the alternating air function remotely. This app (using digital technology) could potentially be an effective intervention for optimizing efficiency in the prevention and treatment of pressure injuries. However, digital technology is not always as useful as intended.

### Prior Work

Digital technology has been studied comprehensively, and according to a barrier analysis published by Mathijssen et al [9], there is a mismatch between the available digital technology and the adoption of digital technology. In this analysis, a scoping review and a survey were conducted, and the barriers and facilitators were classified according to the capability, opportunity, motivation, and behavior (COM-B) model by Michie et al [10]. Most barriers in the analysis were found in the opportunity domain (eg, technical issues). Health care workers reported that digital technology should provide support in delivering health care instead of replacing it. The accessibility and reliability of digital technology were identified as facilitators in the adoption of digital technology. The privacy and security of patient data, training of health care workers, and practical support regarding digital technology were also marked as facilitators [9].

Barriers and facilitators regarding the adoption of digital technology in health care practices have been identified. It is currently unclear whether the Mercury Advance SMARTcare solution can support health care workers in their daily practices. DHG’s hybrid mattress (Mercury Advance) has proven to be clinically efficient in hospital settings [7]. However, the expectations of health care workers will determine whether the Mercury Advance SMARTcare solution is suitable for adoption before clinical effectiveness can be investigated. The Mercury Advance SMARTcare unit is currently being tested at several sites in the United Kingdom, and the effectiveness of the app thus far is unknown. To reveal preconceptions, a study with an explorative design is suitable. Consequently, a generic qualitative study to investigate the expectations of health care workers of the Mercury Advance SMARTcare solution connected to the Mercury Advance mattress is necessary.

### Objective of This Study

The objective of this study is to gain insight into health care workers’ expectations of factors that could hamper or support the adoption of the Mercury Advance SMARTcare unit connected to a Mercury Advance mattress to prevent patients from developing pressure injuries in hospitals and long-term care facilities. The results can be used to improve the implementation process of the Mercury Advance SMARTcare solution and help identify potential knowledge gaps.

## Methods

### Study Design

A generic qualitative study with health care workers was conducted between February and December 2022 using in-depth individual interviews and a focus group. The focus group provided interaction between the participants, which is especially suitable for explorative research [11]. Individual interviews may reveal sensitive concepts, which may be left undiscussed in focus groups. In addition, the results of the individual in-depth interviews confirmed the findings of the focus group and contributed to method triangulation [12].

### Setting

#### Sampling Technique

A purposive sampling technique was adopted for the recruitment of health care workers in both the United Kingdom and the Netherlands. The objective was to gain insight into health care workers’ expectations of the Mercury Advance SMARTcare unit connected to the Mercury Advance mattress. More information about the Mercury Advance SMARTcare solution can be found on the developer’s web page [8].

#### Eligibility

Most of the time, initial contact with study sites was maintained by a DHG product specialist. A team manager of the potential participants had initial contact by email consistently throughout the study. The eligibility criteria were adopted to ensure that participants were able to provide meaningful insights regarding the subject of this study. The required inclusion criteria for the participants were being a registered nurse, physiotherapist, or occupational therapist; working with patients directly; dealing with pressure injury prevention or treatment; and being able to read, write, and speak English or Dutch. When a participant agreed to participate, they would receive a participant information sheet, and an interview with them would be scheduled. Besides the purposive sampling technique, a snowballing selection strategy was used, signifying that the researcher asked the participants included if they knew the potential participants who met the abovementioned eligibility criteria.

#### Domain

The occupational sites of the participants included general hospital wards, psychiatric wards, and a rehabilitation center located in the Netherlands and the United Kingdom. The study population can be considered homogeneous, with health care workers working on preventing and managing pressure injuries. To achieve data saturation in a study, according to Holloway and Wheeler [13], a sample size of 6 to 8 participants is considered sufficient in a homogeneous sample. That is why we included a total of 14 participants. Of these 14 participants, 6 (43%) were scheduled for the focus group interview and the remaining 8 (57%) were scheduled for the individual interviews.

### Data Collection

Qualitative data were collected using a focus group and in-depth individual interviews, which were recorded and transcribed verbatim. Sessions were held face-to-face at a location picked by the participants or via Microsoft Teams (version 1.4.00.22472; Microsoft Corp). Audio recordings were the main source of data. An interview guide for the focus group and the individual interviews was put together beforehand to facilitate a semistructured approach.

To make the participants feel comfortable, the interview started with the following question: “On a scale from 0-10, how important is pressure injury prevention for patients in general?” This question aimed to encourage the participants to narrate their views on patient care and to remember the importance of their work. Subsequently, our interview guide included the following topics, which were discussed using open-ended questions: (1) pressure injury equipment and current procedures (eg, “What is your experience with the pressure ulcer prevention tools that are currently in use in your department?”), (2) technology in health care, (3) SMARTresponse app video, (4) expectations of the SMARTresponse app, and (5) training regarding the SMARTresponse app (eg, “In your opinion, what is required in order to use the SMARTresponse application?”). Our interview guide topics were based on the barriers and the facilitators as described by Mathijssen et al [9]. During the interview, the participants were shown a short video (3 min) of the Mercury Advance SMARTcare solution to ensure that they could vividly imagine the application in their work environment. In the video, a trainer explained the use of the Dyna-Form SMARTresponse control unit and its connectivity to an Apple iPad. The activation of the automatic and manual functions was demonstrated on the iPad, using an app, including how it was applied to the control unit.

The interview guide was pilot-tested with 2 occupational therapists and 2 nursing science students at Utrecht University. A pilot interview was performed in English to improve its feasibility, as the interviewer was not a native English speaker. This gave the novice researcher (JS) the opportunity to get comfortable with the interview guide and to test the amount of content-specific information included in the interview guide. All individual interviews were carried out by JS. The focus group was facilitated by JS and moderated by a second researcher (TvH), who has experience in qualitative research. During the individual interviews and the focus group meeting, observational notes were made to gather nonverbal aspects regarding the data collection and to enhance the credibility of the findings [13].

### Data Analysis

The data were analyzed by 2 independent researchers (JS and TvH) and was based on thematic analysis, as described by Braun and Clarke [14]. Considering the data analysis, an inductive (data-driven) approach was chosen. Data collection and analysis were performed simultaneously to contribute to the constant comparison approach [12]. The audio recordings were transcribed verbatim by JS, and these transcriptions were checked for inconsistencies by TvH. To support the data analysis, ATLAS.ti software (version 22; ATLAS.ti Scientific Software Development GmbH) was used. Entire transcripts were read and reread to get familiar with the data. The initial coding was done by JS and carried out inductively. Codes were discussed with TvH, and a code list was put together. Subthemes were generated to collate all codes from the code list. During this process, a mind mapping approach was used to get familiar with the structure of the data. All codes were run to determine whether they were associated with multiple subthemes. The 2 researchers discussed overarching candidate themes, subthemes, and related codes. During this process, insights were gathered, and the interview guide was adjusted accordingly. Factors emerged from the data, which were summarized by JS, who added illustrative quotes. Next, these factors were confirmed by TvH. Factors were divided into barriers and facilitators according to the objective of the study.

For further involvement in the underlying process of the data analysis, expert validation of preliminary findings was performed by a tissue viability nurse specializing in wound care and management. The findings were acknowledged and presented to the participants for a member check to enhance the credibility and validity of the study [13].

### Ethical Considerations

This study was conducted in accordance with the General Data Protection Regulation [15] and the Declaration of Helsinki [16]. All participants provided informed consent before the study. The participants of the study were not subjected to procedures, actions, or behavioral rules. The expectations of health care workers were the primary study parameters, which fall outside the scope of medical or scientific research. According to the Central Committee on Research Involving Human Subjects, this study does not apply to the Medical-Scientific Research with People Act and was therefore not reviewed by a Medical Research Ethics Committee [17]. In addition to the interview, participants’ characteristics were recorded to describe the study population. Participant identification codes were generated to ensure the participants’ anonymity. Transcribed interviews, signed consent forms, participant identification codes, and study metadata were all stored on the university’s research drive (HU University of Applied Sciences) [18], which is designed for the handling and storage of research data. This cloud service acquired the International Organization for Standardization 27001 certification and, therefore, legally adheres to the General Data Protection Regulation [15].

## Results

### Demographics

A total of 18 participants agreed to participate in the study, however, 4 (22%) were not able to schedule an interview with the researchers or attend the focus group meeting. Of the 14 participants included in the study, 6 (43%) attended the focus group and 8 (57%) participated in the individual interviews. One (7%) interview was carried out with a participant working in the United Kingdom; the remaining interviews and the focus group meeting were held with participants working in the Netherlands. The duration of the individual interviews was 32 to 67 minutes, with a mean interview time of 51 (SD 11) minutes. The duration of the focus group meeting was 96 minutes. The characteristics of the participants are presented in Table 1.

**Table 1:** Demographic characteristics of the participants (N=14).

Participant ID	Age (y), range^a^	Data collection method	Duration (min)	Occupation	Work experience (y)	Highest educational level
P1	30-39	Interview	32	Tissue viability nurse	14	HPE^b^ nursing
P2	30-39	Focus group	96	Nurse	12	SVE^c^ nursing
P3	60-69	Focus group	96	Physiotherapist	40	HPE physiotherapy
P4	60-69	Focus group	96	Nurse	36	SVE nursing
P5	30-39	Focus group	96	Nurse	14	SVE nursing
P6	50-59	Focus group	96	Nurse	35	SVE nursing
P7	50-59	Focus group	96	Nurse	32	SVE nursing
P8	40-49	Interview	67	Tissue viability nurse	25	SVE nursing
P9	20-29	Interview	56	Nurse	5	HPE nursing
P10	20-29	Interview	50	Nurse	1	HPE nursing
P11	30-39	Interview	63	Nurse	7	HPE nursing
P12	20-29	Interview	47	Nurse	3	HPE nursing
P13	20-29	Interview	49	Tissue viability nurse	8	HPE nursing
P14	40-49	Interview	45	Tissue viability nurse	27	SVE nursing

^a^Age is presented as a range to ensure participants’ anonymity.

^b^HPE: higher professional education.

^c^SVE: secondary vocational education.

### Overview

In total, 13 factors were identified that could hamper or support the adoption of the Mercury Advance SMARTcare solution. These 13 factors were included in the 22 subthemes as identified during the first phases of the qualitative analysis. The 22 subthemes were collated into four overarching themes: (1) *factors specifically related to SMARTresponse,* (2) *vision on innovation,* (3) *match with health care activities, and* (4) *materials and resources involved.* An overview of the themes, subthemes, and related factors is presented in Multimedia Appendix 1. During the final phases of the qualitative analysis, the 13 factors were divided into barriers or facilitators. To ensure that data saturation was achieved in the analysis of the study, 2 individual interviews were conducted after the division into barriers and facilitators. Analysis of these last interviews did not provide further insights into the barriers or facilitators overview. Using illustrative quotes, the Multimedia Appendix 2 shows how the factors can hamper (barriers) or support (facilitators) the adoption of the Mercury Advance SMARTcare solution.

### Factors Specifically Related to SMARTresponse

The factors specifically related to SMARTresponse included three subthemes: (1) SMARTresponse, (2) training, and (3) supplier. Six facilitators were identified within these subthemes (reference to *Q* in the text refer to specific quotes in Multimedia Appendix 2). The Mercury Advance SMARTcare solution may have a signaling function (Q1-Q3) or a preventing function before pressure injuries occur (Q4-Q6). The adoption of the app is conditional upon the patient category (Q13-Q16). Patient involvement may help gain insight into patient movement, but, as a result, evaluation of adherence to the app is necessary (Q11 and Q12). Real-life practice with the app and available representatives with knowledge of the app are facilitators for both training and implementation of the system (Q23-Q30).

Four barriers were identified. The system will most likely not be suitable for a psychiatric ward (Q7 and Q8). The lights on the pump unit were a point of interest, especially at night (Q20-Q22). Although participants said that the supplier is required to manage the training (Q32), maintaining contact with the ward by the supplier was said to be undesirable (Q31). Finally, this app would require a lot of effort and persistence in the beginning, and the added value of it was discussed (Q17-Q19).

### Vision on Innovation

The vision of the health care workers on innovation included six subthemes: (1) vision on pressure injuries, (2) adoption of innovation, (3) vision on technology, (4) pressure injury impact and present performance, (5) reflection on self, and (6) remote health care*.* Three facilitators were identified within these subthemes. The app could serve as a preventive aid (Q42 and Q43), and it could support the health care workers by acting as a signaling function (Q33 and Q34). The nature of the introduction of the system may help encourage the health care workers to use it (Q38-Q40).

Two barriers were identified. The app does not appear to be suitable for patient involvement in the neurological patient category (Q41). Participants’ perception of patient repositioning changes whenever a pressure injury unit is adopted. Patient repositioning is less prioritized or even considered redundant (Q35-Q37 and Q44-Q46).

### Match With Health Care Activities

The match with health care activities included six subthemes: (1) patient factors, (2) nurses’ tasks, (3) patient repositioning, (4) patients’ comfort, (5) mattress change, and (6) hygiene. A total of 4 facilitators were identified within these subthemes. The app can be useful whenever the patient’s movement is unknown (Q47). Patient involvement results in control (Q48 and Q49). Cutoff values can determine the adoption of the app (Q52-Q54). The app can save a lot of time and effort (Q56 and Q57).

Five barriers were identified. A psychiatric ward may not be a suitable environment for the app (Q51 and Q52). Unlike the last facilitator described in the previous paragraph, the app requires new tasks as well (Q55). The frequency of patient repositioning is not clear, resulting in a debate among colleagues, especially when mattresses are used (Q58 and Q59). A mattress change is often performed during the work shift and does not require much effort for the health care workers (Q62 and Q63). Finally, some participants described that a hybrid mattress feels hard (Q60).

### Materials and Resources Involved

The materials and resources involved included seven subthemes:(1) pressure injury equipment, (2) organization, (3) dynamic support surfaces, (4) performance appliances, (5) financials, (6) devices, and (7) time. Three facilitators were identified. In most organizations, representatives with specific areas of interest are present, which could help support the implementation of the system (Q66-Q68). In some centers, devices compatible with the app were readily available (Q73 and Q74). Patients experienced a dynamic mattress to be less comfortable than a hybrid mattress, resulting in supportive opinions about the Mercury Advance SMARTcare solution (Q69 and Q70).

Four barriers were identified. Some participants said that a dynamic mattress with configurable settings felt more comfortable than a hybrid mattress (Q64). The performance of appliances, such as the wireless network, needs to function sufficiently for the app to run properly (Q71 and Q72). At times, there was no access to devices compatible with the app. Participants described the adoption of a personal smartphone as undesirable, which can be considered a barrier (Q75 and Q76). Finally, the adoption of the system requires effort and time, which are not always available (Q77 and Q78).

## Discussion

### Principal Findings

This study found 13 factors that could hamper or support the adoption of the Mercury Advance SMARTcare solution. Factors from four overarching themes were included: (1) factors specifically related to SMARTresponse, (2) vision on innovation, (3) match with health care activities, and (4) materials and resources involved*.* Factors were often identified as either a facilitator or a barrier, but occasionally a factor was identified as both. This was the case with the following factors: *patient involvement, implementation engagement, time consuming, accessibility to devices compatible for the app,* and *comfort*. *Signaling function, SMARTresponse as prevention, patient category, representatives,* and *implementation strategy* were identified as facilitators. *Perception toward patient repositioning, accessibility to pressure injury aids,* and *connectivity* were identified as barriers.

This explorative study identified several factors that seem to influence the adoption of the Dyna-Form SMARTresponse app, according to the expectations of health care workers. The Mercury Advance SMARTcare solution could help support health care workers in their daily practices as a preventive aid with certain conditions in mind.

Patient involvement may serve as a facilitator, which was unknown according to the brochure for the Mercury Advance SMARTcare solution [8]. Therefore, patients need to be involved in the training aspect of the system whenever this is possible.

Before the implementation of the system is commenced, preliminary conditions apply. A guideline specifying the patient category or facility for which the system is suitable is needed. Health care workers are required to have and be able to operate a smartphone or tablet; otherwise, the app cannot be operated. Finally, the wireless network must function properly to make the app run smoothly and, as a result, reduce the risk of health care workers feeling agitated about the performance of the app.

Whenever the system is adopted in health care settings, training the health care workers is an important aspect of enhancing the success rate. Representatives from a specific area of interest need to engage more in training to ultimately support other health care workers and to act as an early adopter. Practical training in which the health care workers can experiment with the Mercury Advance SMARTcare solution could enhance the proportion in which the health care workers will adopt the system.

Although this study has identified barriers and facilitators that can hamper or support the adoption of the Mercury Advance SMARTcare solution, a knowledge gap still remains regarding health care workers who work in home care nursing, as these workers were not included in the study population. Therefore, more research on health care workers working in home care nursing is necessary to acknowledge the findings of this study. Subsequently, an implementation project is recommended for the promotion of the app in health care facilities and to determine its effectiveness regarding pressure injury prevention.

### Comparison to Prior Work

During the analyses, similarities were observed between the subthemes and the diffusion of innovation theory [19]. This theory describes five categories of adopters in the context of technological adoption: (1) technology enthusiasts, (2) visionaries, (3) pragmatists, (4) conservatives, and (5) skeptics. The theme *vision on innovation* demonstrates the participants’ preconceptions of digital technology and their views on the adoption of the SMARTresponse app. A division was observed among the participants, with some being obvious skeptics and others appearing to be visionaries or technology enthusiasts. In addition, the diffusion process among colleagues, as explained by the participants, clearly emerged from the data. Most participants reported that enthusiastic colleagues or representatives play a crucial role in the adoption process of a new product, practice, or idea.

Several studies have investigated the adoption of sensors to detect patient movement and increase adherence to patient repositioning protocols [20-23]. All studies reported that adherence to turning protocols increased whenever a sensor was adopted in intensive care units. According to the study by Yap et al [23], participants expressed satisfaction with the monitoring system and recommended improvements to support the adoption and use of technology. Our study included participants working at hospitals, psychiatric wards, and a rehabilitation center, which are considerably different from an intensive care unit. Moreover, the patient sensors that were adopted in the previous studies [20-23] are not comparable with the Mercury Advance SMARTcare solution, which uses a control unit to detect patient movement. However, qualitative outcome measures from a previous study [23] are in line with the findings of this study, and quantitative measures from previous studies [20-23] suggest that health care workers’ awareness of a patient’s movement or nonmovement increases when sensors are adopted.

### Strengths and Limitations

An important strength of this study was the inclusion of 4 tissue viability nurses and 1 physiotherapist instead of nurses only. All these health care workers worked with pressure injury aids and cared for patients with pressure injuries on a regular basis. That is how it was possible to identify an extensive scope of perspectives from health care workers with different opinions regarding pressure injury prevention and treatment. An additional factor was identified during the analysis of the 12th transcript, with data saturation not being confirmed at first. For that reason, 2 more individual interviews were conducted to confirm data saturation and identify themes and factors regarding the first 12 transcripts. Furthermore, preliminary findings were presented repeatedly in a research group with experienced researchers, which enhanced the confirmability of the findings [13].

This study also has limitations. The initial interview with participant 1 had a duration of 32 minutes, which is relatively short compared to the other interviews. However, the subthemes identified in the first interview were also present in the other interviews. Although the occupational sites are considered heterogeneous, home care nursing was not incorporated as a study population site. Therefore, the perspectives of health care workers who work in home care are not incorporated in this study, despite them being an interest group according to the National Pressure Injury Advisory Panel [24]. Furthermore, in focus groups, participants may not contribute equally, leaving opinions and views on a specific topic undiscovered. When conducting focus groups in addition to individual interviews, different concepts might have been identified compared to individual interviews alone. However, we believe that conducting individual interviews felt inaccurate in this exploratory study because participant interactions would not have been revealed. Finally, the data collection for this study was conducted by a novice researcher (JS) with limited experience in qualitative research. To overcome this limitation, a second researcher with noticeable experience in qualitative research checked the first transcripts of the recorded interviews to confirm content validity.

### Conclusions

This study explored the expectations of factors that could hamper or support the adoption of the Mercury Advance SMARTcare unit connected to a Mercury Advance mattress to prevent patients from developing pressure injuries in hospitals and long-term care facilities. The system is developed to support health care workers in their daily practices, especially as a preventive aid and due to its signaling function. However, several conditions need to be met to enhance the adoption of the system, such as guidelines concerning adherence to patient repositioning, the engagement of representatives in training, and a reliable wireless network. The factors identified in this study can be used to facilitate the implementation process and adoption of the Mercury Advance SMARTcare solution and to help provide quality care to patients who are at risk of developing pressure injuries.

## Appendix

Multimedia Appendix 1 Themes (bold), subthemes (underlined), and related factors (italics) regarding the Dyna-Form SMARTresponse app. Some factors were related to multiple themes or subthemes.

Multimedia Appendix 2 Barriers and facilitators regarding the Dyna-Form SMARTresponse app.

## Abbreviations

COM-B: capability, opportunity, motivation, and behavior
DHG: Direct Healthcare Group

## Data Availability

The data sets generated during and analyzed during this study are not publicly available since the participants of this study did not provide written consent for their data to be shared publicly.
