# Supplementary figures and images for: Health Care Workers’ Expectations of the Mercury Advance SMARTcare Solution to Prevent Pressure Injuries: Individual and Focus Group Interview Study
Source: JMIR Nurs. 2024 Apr 18;7:e47992. doi: 10.2196/47992 (PMC11066743; doi:10.2196/47992)

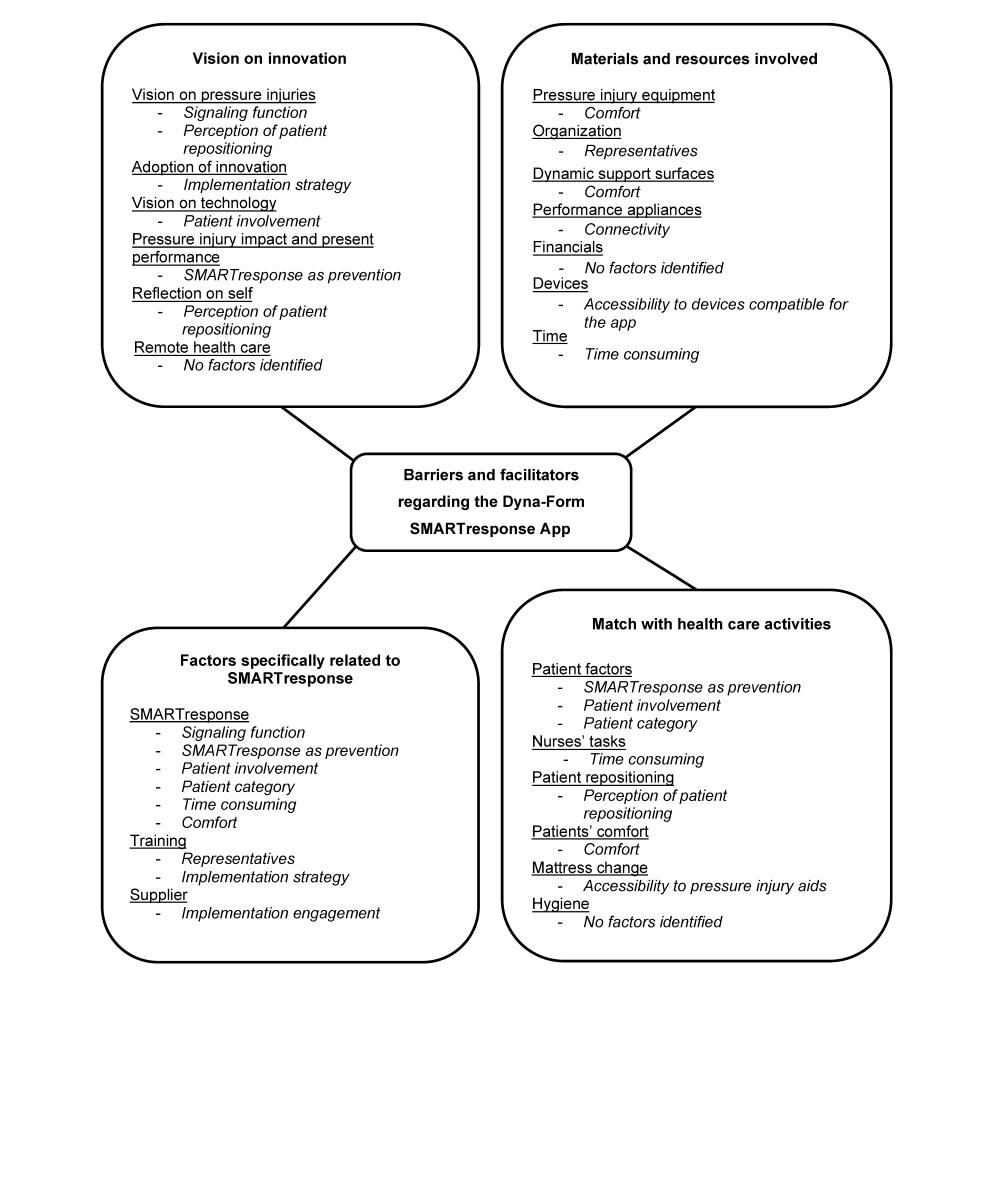

Supplement: Multimedia Appendix 1 [file nursing_v7i1e47992_app1.png]
