# Supplementary material for: Health Care Workers’ Expectations of the Mercury Advance SMARTcare Solution to Prevent Pressure Injuries: Individual and Focus Group Interview Study
Source: JMIR Nurs. 2024 Apr 18;7:e47992. doi: 10.2196/47992 (PMC11066743; doi:10.2196/47992)
Supplement: Multimedia Appendix 2 [file nursing_v7i1e47992_app2.docx]

**Multimedia Appendix.** Barriers and facilitators regarding the Dyna-Form SMARTresponse app.

| Themes | Subthemes (frequency of quotes) | Factors^a^ | Barrier or facilitator | Illustrative quotes^b^ |
| --- | --- | --- | --- | --- |
| Factors specifically related to SMARTresponse | SMARTresponse (100) | Signaling function | - Facilitator - Facilitator - Facilitator | - Q1: “Patients with paralysis, they are barely able to reposition themselves in bed, awareness about the movement is a good thing. So, I am excited about the signaling function.” [P9] - Q2: “Well perhaps, awareness regarding the patient movement is obtained with this smart system, that patients reposition themselves a lot less than we assume.” [P11] - Q3: “A little more, smart technology...on a regular mattress...so we can detect in advance.” [P7] |
| Factors specifically related to SMARTresponse | SMARTresponse (100) | SMARTresponse as prevention | - Facilitator - Facilitator - Facilitator | - Q4: “Uhm, I think uhm, preventing pressure ulcers from developing, uhm, is uhm, y-y-y-y-your highest concern(...) so prevention is really important.” [P14] - Q5: “Because if it is set to automatic mode... the mattress itself would... identify the patient hasn’t repositioned for so long... and... do the... (...) that is the main benefit I would see from this.” [P1] - Q6: “Uhm, but more in the context of prevention. Perhaps, if we can start earlier on, to prevent a patient from developing pressure injuries.” [P11] |
| Factors specifically related to SMARTresponse | SMARTresponse (100) | Patient involvement | - Barrier - Barrier - Facilitator - Facilitator - Facilitator - facilitator | - Q7: “The system positioned to the bed(...) it will appeal to other patients to press these buttons.” [P2] - Q8: “ (...) it does not apply to all patients, some patients will demolish the system (...)”[P6]; - Q9: “They simply do not acquire the system.”[P5]; - Q10: “No, they do not acquire the system.” [P6] - Q11: “Experience it yourself and evaluate afterwards, we can decide to switch to automatic mode eventually, but you should give it a try first.” [P9] - Q12: “I believe it can gain insight for them, how often they reposition themselves(...)but it is essential that we explain the employment of the application (...)” [P11] |
| Factors specifically related to SMARTresponse | SMARTresponse (100) | Patient category | - Facilitator - Facilitator - Facilitator - Facilitator | - Q13: “In a hospital it would...be optimal.” [P2] - Q14: “Yes. I believe uhm, it is it depends on the patient, at all.” [P9] - Q15: “ (...)Sometimes, one can decide that employment of the system is not suitable for a specific patient.” [P12] - Q16: “I think that uhm, the geriatric ward is going to be really happy with this!” [P14] |
| Factors specifically related to SMARTresponse | SMARTresponse (100) | Time consuming | - Barrier - Barrier - Barrier | - Q17: “A lot of time...effort...and persistence.” [P7] - Q18: “Yes. If I am imagining, I am wondering, what is the added value?” [P6] - Q19: “But I believe that it requires a lot of effort in the beginning.” [P10] |
| Factors specifically related to SMARTresponse | SMARTresponse (100) | Comfort | - Barrier - Barrier - Barrier | - Q20: “It is like disco-lights(...)In my opinion the design would be different.” [P8] - Q21: “Start and the other functions, does it generate much light?” [P9] - Q22: “Yes, I can really imagine covering the lights with a towel, because the illumination at night (...)” [P9] |
| Factors specifically related to SMARTresponse | Training (79) | Representatives | - Facilitator - Facilitator - Facilitator | - Q23: “Target at the involvement of representatives specifically(...)Then, uhm...training on the job.” [P8] - Q24: “Representatives regarding pressure injuries, uhm, do the training. So that they can facilitate the training to us.” [P9] - Q25: “In our center we actually try to facilitate the representatives-training by representatives themselves, we assessed all matrasses during this moment recently.” [P13] |
| Factors specifically related to SMARTresponse | Training (79) | Implementation strategy | - Facilitator - Facilitator - Facilitator - Facilitator - Facilitator | - Q26: “Yes, start with one person and... if...” [P6]; - Q27: “Perhaps one system for each ward?” [P5] - Q28: “So, I guess, real-life practice will benefit the most, so the system needs to be present on the ward.” [P9] - Q29: “I assume that people want to receive information about the added value, the efficiency of the system.” [P12] - Q30: “So, what he did back then, which I believed suited just fine. He installed the system itself on the ward. He did not prepare a clinical education specifically, but more like a practical lesson(...) whenever I push this button, the following happens.” [P14] |
| Factors specifically related to SMARTresponse | Supplier (20) | Implementation engagement | - Barrier - Facilitator | - Q31: “In our center these kinds of tasks are performed by the technical department, so whenever they are in close contact with the supplier it is ok. The wards do not necessarily need to be in contact with the supplier(...)” [P10] - Q32: “One can decide to invite the supplier, for like 30 minutes, twice a week at first... and then reduce the number of visits.” [P9] |
| Vision on innovation | Vision on pressure injuries (58) | Signaling function | - Facilitator - Facilitator | - Q33: “I believe whenever a certain system can help a health care worker by indicating that the patient hasn’t repositioned for too long(...)it is obtained positively, regardless of the health care workers’ attitude toward a specific system.” [P8] - Q34: “Well, to prevent is always better than to cure(...)If it can be prevented so to say, then you will not be bothered...I do not have to be concerned about injuries or infections.” [P10] |
| Vision on innovation | Vision on pressure injuries (58) | Perception of patient- repositioning | - Barrier - Barrier - Barrier | - Q35: “And then we conduct patient repositioning in the evening. I know it is intended, to, to reposition, I am aware of that. But in some sort of way, it is less prioritized, I consider.” [P9] - Q36: “I was thinking, if the mattress can prevent that...you should perform patient repositioning(...)Then it can be excellent.” [P6] - Q37: “Because I believe it can be dangerous whenever one starts to assume Ahaaa, this technology is wonderful, the pump is delivering all the work, so I am going to let the pump do all the work...you get that?” [P13] |
| Vision on innovation | Adoption of innovation (40) | Implementation strategy | - Facilitator - Facilitator - Facilitator | - Q38: “You want to encourage them by demonstrating, and working with it properly. And then, the want to engage, eventually.” [P9] - Q39: “Yes, I believe that, whenever it is introduced properly, they will be very enthusiastic about it.” [P10] - Q40: “At first most of the colleagues were pessimistic about it(...) I think that actually uhm, the best example is that you can come to a breaking point as long as you believe in it yourself.” [P13] |
| Vision on innovation | Vision on technology (34) | Patient involvement | - Barrier | - Q41: “But we work with patient category with neurological disfunctions, they need to understand...how everything works as well.” [P9] |
| Vision on innovation | Pressure injury impact and present performance (45) | SMARTresponse as prevention | - Facilitator - Facilitator | - Q42: “I assume we should actually start, whenever the first symptoms develop, just preventively.” [P3] - Q43: “I am still concerned about the employment of aids, in my opinion they are adopted too late.” [P7] |
| Vision on innovation | Reflection on self (17) | Perception of patient repositioning | - Barrier - Barrier - Barrier | - Q44: “I assume the mattress has some added value. It serves a purpose, so, the awareness towards patient repositioning is less, despite the know-how, that it is important.” [P9] - Q45: P10: “Uhm...and these patients meet the requirements to employ the pro-mat.” Researcher: “Ok, and uhm, then you don’t perform the patient repositioning every four hours?”; P10: “No.”; Researcher: “Because of the pro-mat?”; P10: “Yes.” [P10] - Q46: P13: “Meaning they, you are trying to say that they think like; a patient is subjected to a mattress with a pump unit, now I don’t have to perform patient repositioning?”; JS: “Hm-hm.”; P13: “A lot!” [P13] |
| Vision on innovation | Remote health care (11) | No factors identified | No barrier or facilitator identified | No illustrative quote identified |
| Match with health care activities | Patient factors (97) | SMARTresponse as prevention | - Facilitator | - Q47: “But our ward, as it is specifically for intake, it is convenient as I am not familiar with the patient.” [P5] |
| Match with health care activities | Patient factors (97) | Patient involvement | - Facilitator - Facilitator - Barrier - Barrier | - Q48: “On a patients’ unit(...)they want to be in control themselves and they want to keep it that way.” [P9] - Q49: “Yes, absolutely yes.(...)I believe it is important, uhm, whenever one is aware of the situation. A patient needs to be involved.” [P10] - Q50: “We care for psychiatric patients(...) They are going to molest the system, make it dysfunctional.” [P2] - Q51: P2: “Yes, but the thing positioned on the bed, it... on which one can operate the functions.” Researcher: “Yes...” P2: “It is attractive(...) to press these buttons.” [P2] |
| Match with health care activities | Patient factors (97) | Patient category | - Facilitator - Facilitator - Facilitator | - Q52: “And then cut-off values can determine whether a bed is employed to certain patients.” [P9] - Q53: “Uhm, no I believe with uhm, with certain patients uhm, the ones who are bed-bound. In that can be very useful.” [P10] - Q54: “Yes, in a hospital this can be, really beneficial.” [P2] |
| Match with health care activities | Nurses’ tasks (33) | Time consuming | - Barrier - Facilitator - Facilitator | - Q55: “Yes, and sometimes there are a lot of new tasks, it is not possible to keep up with that, you receive e-mails of everything, you just can’t keep up with it.” [P9] - Q56: “So, the only thing one should do is connect the pump, yes that would save a lot of time and effort.” [P10] - Q57: “Because then the system is becoming more and more interesting [SMARTresponse](...)Well, at this time, switching a mattress requires effort(...)For patients it is exhaustive, as it is for us.” [P14] |
| Match with health care activities | Patient repositioning (17) | Perception of patient repositioning | - Barrier - Barrier | - Q58: “Sometimes it causes a debate, should one awake a patient to perform patient repositioning at night. So yes, you arrange these matters with the patient.(...)with the dynamic mattress we notice, you should perform patient repositioning, but it is performed less, in my opinion.” [P9] - Q59: “Because we observe that the patient does not leave the bed and has already developed a pressure injury. We already employ this mattress and uhm, then we perform patient repositioning every two hours, but I’m not sure(...)But it is not a guideline or protocol that we follow.” [P11] |
| Match with health care activities | Patients’ comfort (20) | Comfort | - Barrier - Barrier | - Q60: “It feels harsh (hybrid mattress), I am wondering to myself... what effect does that have to the body.” [P7] - Q61: “Well, in my opinion the patient does not experience disadvantages, whenever the pump is constantly switched on.” [P11] |
| Match with health care activities | Mattress change (12) | Accessibility to pressure injury aids | - Barrier - Barrier | - Q62: “Uh no, they are available, because uh, considering a mattress, there is a specific mattress for every patient on the ward. The other mattresses can be ordered, and then it will arrive on the ward within two hours.” [P9] - Q63: “Uh, and ordering the mattress, the technical department will handle the distribution to the ward.” [P10] |
| Match with health care activities | Hygiene (5) | No factors identified | No barrier or facilitator identified | No illustrative quote identified |
| Materials and resources involved | Pressure injury equipment (48) | Comfort | - Barrier - Barrier | - Q64: “When compared to the air-mattresses, the ones, they were able to be configurated with patients’ weight(...)With these that is not the case.” [P7] - Q65: “Patients are positioned on a regular mattress for a lifetime. A decupré, or whatever they call it. And then, at the moment they are becoming more fragile so to say, then they are subjected to a mattress, that...once again, feels harsh, and then I think to myself...what’s the effect of that to the body?” [P7] |
| Materials and resources involved | Organization (54) | Representatives | - Facilitator - Facilitator - Facilitator | - Q66: “She focuses on the details, investigates our present practice and suggests improvements, whenever this is needed.” [P9] - Q67: “ (...) I believe they arrange meetings together, so for us, we can ask them everything regarding a subject. I think they receive more training concerning a specific topic, when compared to us.” [P12] - Q68: “Yes of course, it is an excellent tool to distribute information in that way.” [P14] |
| Materials and resources involved | Dynamic support surfaces (23) | Comfort | - Facilitator - Facilitator | - Q69: “That was the case as well, with the other plastic air... reposition-mattresses, they were, they were, those were uncomfortable as well.” [P6] - Q70: “Yes, they experience the feeling as if they are floaty, as if they are floating*laughs*.” [P11] |
| Materials and resources involved | Performance appliances (20) | Connectivity | - Barrier - Barrier | - Q71: “(...)they have to go and check how this works, how it would affect on the cloud system, how it affects on the Wi-Fi system, you name it, o my god, the amount of meetings that I have had.” [P1] - Q72: P11: “But it should work.” Researcher: “Oh yes.” P11: “But in my case it did work.”; Researcher: “Oh, so it depends on the Wi-Fi so to say?”; P11: “Yes, yes.” [P11] |
| Materials and resources involved | Financials (22) | No factors identified | No barrier or facilitator identified | No illustrative quote identified |
| Materials and resources involved | Devices (47) | Accessibility to devices compatible for the app | - Facilitator - Facilitator - Barrier - Barrier | - Q73: “It is a pretty big ward, but I guess, twenty devices in total? So, one unit for each nurse.” [P10] - Q74: “Uhm, yeah we have smartphones which are ward-specific to uhm, to run several applications.” [P12] - Q75: “And uhm, I believe the regulations are quite clear in the direction that usage of your own smartphone is undesirable. I will address this to my direct colleagues, as for the doctors.” [P8] - Q76: “Uhm, not at the moment. Let me put it this way, in general no(...) the digital environment does not allow that, at the moment.” [P14] |
| Materials and resources involved | Time (13) | Time consuming | - Barrier - Barrier | - Q77: “If I am being honest, in these crazy times we are in right now, I wouldn’t even be able to look at this application.” [P6] - Q78: “Uh, and at the time everyone found out how to work with it and knows how to use it(...)Uh, especially because there are some colleagues which are older, and they are not that into smartphones. Uhm, and when they are going to receive notification on top of that. Uh-yeah, the, I am not sure about that.” [P10] |

^a^Several factors were found among multiple subthemes.

^b^Illustrative quotes, identified with a code (Q1-Q78), related to the factors are presented in the last column.
